# An Epitope-Specific LGI1-Autoantibody Enhances Neuronal Excitability by Modulating Kv1.1 Channel

**DOI:** 10.3390/cells11172713

**Published:** 2022-08-31

**Authors:** Johanna Extrémet, Oussama El Far, Norbert Ankri, Sarosh R. Irani, Dominique Debanne, Michaël Russier

**Affiliations:** 1 UNIS, INSERM, Aix-Marseille Université, 13015 Marseille, France; 2 Oxford Autoimmune Neurology Group, Nuffield Department of Clinical Neurosciences, Oxford University, Oxford OX3 9DU, UK

**Keywords:** LGI1, Kv1.1, epilepsy, limbic encephalitis, autoantibodies

## Abstract

Leucine-rich Glioma-Inactivated protein 1 (LGI1) is expressed in the central nervous system and its genetic loss of function is associated with epileptic disorders. Additionally, patients with LGI1-directed autoantibodies have frequent focal seizures as a key feature of their disease. LGI1 is composed of a Leucine-Rich Repeat (LRR) and an Epitempin (EPTP) domain. These domains are reported to interact with different members of the transsynaptic complex formed by LGI1 at excitatory synapses, including presynaptic Kv1 potassium channels. Patient-derived recombinant monoclonal antibodies (mAbs) are ideal reagents to study whether domain-specific LGI1-autoantibodies induce epileptiform activities in neurons and their downstream mechanisms. We measured the intrinsic excitability of CA3 pyramidal neurons in organotypic cultures from rat hippocampus treated with either an LRR- or an EPTP-reactive patient-derived mAb, or with IgG from control patients. We found an increase in intrinsic excitability correlated with a reduction of the sensitivity to a selective Kv1.1-channel blocker in neurons treated with the LRR mAb, but not in neurons treated with the EPTP mAb. Our findings suggest LRR mAbs are able to modulate neuronal excitability that could account for epileptiform activity observed in patients.

## 1. Introduction

Autoantibodies directed against Leucine-rich Glioma-Inactivated protein 1 (LGI1) are found in patients with limbic encephalitis (LE) who have frequent focal seizures and hippocampal atrophy as hallmarks of their disease [1,2,3]. Hyperexcitability and epileptiform activities have been recorded in the hippocampi of mice and rats treated with serum from patients with LGI1 autoantibodies, suggesting their direct pathogenicity [4,5]. LGI1 is a soluble molecule composed of a Leucine-Rich Repeat (LRR) and an Epitempin (EPTP) domain. The LRR domain is reported to mediate transsynaptic homo-oligomerization, whereas the EPTP domain allows for LGI1 to dock on its pre- and post-synaptic receptors, ADAM23 and ADAM22, respectively [6]. Serum LGI1 antibodies have been shown to target both the LRR and EPTP domains of LGI1. Sera have been shown to cause both a down-regulation of two interaction partners of LGI1: the presynaptic voltage-gated potassium channel Kv1 and the postsynaptic AMPA receptors [4,5,7,8]. Furthermore, polyclonal serum antibodies can prevent the interaction of LGI1 with ADAM22 and potentiate excitatory glutamatergic synapses [7,8,9].

More recent reports aimed to better characterize the relative pathogenic roles of domain-specific antibodies by studying recombinant monoclonal LGI1 autoantibodies (mAb) derived from patient B cells [7,8]. mAbs targeting the LRR domain were shown to internalize the LGI1 protein after it docked to ADAM22/ADAM23 receptors [8]. In contrast, EPTP-mAbs operated by competing with LGI1 for binding to ADAM22 [7,8]. Both mAb categories were shown to modulate neuronal excitability, but LRR-mAbs induced more robust effects and behavioral deficits [7,8].

In order to directly compare the electrophysiological effects of LRR- and EPTP-mAbs, we measured intrinsic excitability of CA3 neurons after their application. As LGI1 can alter the inactivation gating of the Kv1 channels [10], which tune neuronal excitability in CA3 pyramidal neurons [11], we tested whether blocking the Kv1.1 subunit with a specific antagonist, dendrotoxin-k (DTx-k), affected intrinsic excitability, which could be abrogated by the mAbs.

## 2. Materials and Methods

**Rat hippocampal slice cultures:** All experiments were carried out according to the European and Institutional guidelines for the care and use of laboratory animals (Council Directive 86/609/EEC and French National Research Council) and approved by the local health authority (D13055-08, Préfecture des Bouches-du-Rhône). Slice cultures were prepared as described previously [12]. In brief, young Wistar rats (P7–P10) were anesthetized with isoflurane and killed by decapitation, the brain was removed, and each hippocampus was dissected. Hippocampal slices (350 μm) were obtained using a Vibratome (Leica, VT1200S, Wetzlar, Germany). They were placed on 20 mm latex membranes (Millicell) inserted into 35 mm Petri dishes containing 1 mL of culture medium and maintained for up to 8 days in an incubator at 34 °C, 95% O_2_–5% CO_2_. The culture medium contained 25 mL MEM, 12.5 mL HBSS, 12.5 mL horse serum, 0.5 mL penicillin/streptomycin, 0.8 mL glucose (1 M), 0.1 mL ascorbic acid (1 mg/mL), 0.4 mL Hepes (1 M), 0.5 mL B27, and 8.95 mL sterile H_2_O. To limit glial proliferation, 5 μM Ara-C was added to the culture medium starting at 4 d in vitro (DIV).

**Figure 1:**
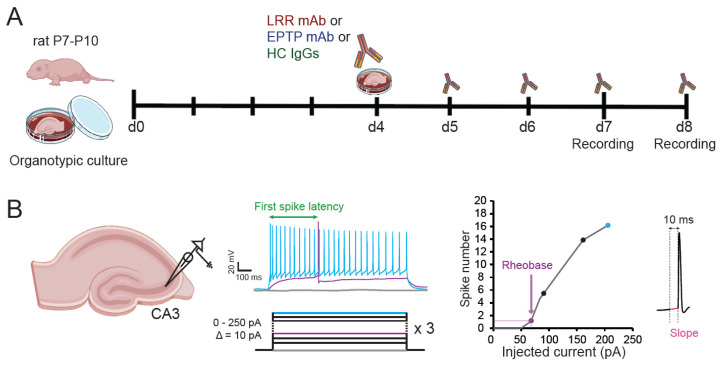
Experimental procedures. (**A**) Timeline representing the patient-derived mAb incubation protocol. Hippocampal slices of rat aged of 7 to 10 post-natal days (P7-P10) were put in culture and individually incubated with either a recombinant LRR-mAb, a recombinant EPTP-mAb, or polyclonal IgGs from healthy human patient (HC IgGs) at 4 days of culture until the days of experiment at day 7 (d7) and day 8 (d8). (**B**) CA3 pyramidal neurons from organotypic cultures treated with the different antibodies were recorded in current-clamp mode. The number of spikes elicited by the injection of depolarizing currents with increments of 10 pA from 0 to 200 pA was measured to obtain an input–output curve. The minimal current injected to elicit at least an action potential (rheobase) was determined. The slope of the membrane potential trajectory before the action potential was quantified in a time window of 10 ms.

**Application of the mAbs:** LGI1-specific recombinant mAbs were produced as described in Ramberger et al. 2020 [8]. Polyclonal IgGs purified from total healthy human serum (Protein G Sepharose 4 Fast Flow, 17-0618-01, Cytiva, Marlborough, MA, USA) were used as a control (Human Control: HC). The electrophysiological effect of one recombinant LRR-mAb (mAb01, [8]) and one EPTP-mAb (mAb6212, [8]) were assessed individually in rat CA3 pyramidal neurons. HC IgGs and LGI1-mAbs were applied daily from the 4th day in vitro (DIV4) until the day of the experiment, at a final concentration of 4 ng/µL in culture medium, in direct contact with the hippocampal organotypic cultures (Figure 1A).

**Electrophysiology:** Whole-cell recordings were obtained from CA3 neurons in organotypic cultures at DIV7 and DIV8. Recordings were carried out in a limit of 6 h after the last application of the IgGs. The external solution contained: 125 mM NaCl, 26 mM NaHCO_3_, 3 mM CaCl_2_, 2.5 mM KCl, 2 mM MgCl_2_, 0.8 mM NaH_2_PO_4_, and 10 mM D-glucose, and was equilibrated with 95% O_2_–5% CO_2_. Patch pipettes (7–10 MΩ) were filled with a solution containing (mM): K-gluconate 120, KCl 20, Hepes 10, EGTA 0.5, MgCl_2_ 2, Na_2_ATP 2, and NaGTP 0.3 (pH 7.4). Liquid junction potential (-12 mV) was not compensated. Organotypic cultures were quickly washed in the perfused recording chamber with the external solution by increasing the speed of perfusion pumps to facilitate the patch-clamp procedure. All recordings were made at 29 °C in a temperature-controlled recording chamber (Luigs & Neumann, Ratingen, Germany). CA3 pyramidal neurons were recorded in current clamp with a Multiclamp 700B Amplifier (Axon Instruments, Molecular Devices). Excitability was measured by delivering a range of long (1 s) depolarizing current pulses (10–200 pA, by increments of 10 pA) and counting the number of action potentials (Figure 1B). Ionotropic glutamate and GABA_A_ receptors were blocked with 2–4 mM kynurenate and 100 μM picrotoxin, respectively. Input–output curves corresponding to the number of action potential elicited by each increment of injected current were determined for each neuron and two parameters were examined; the rheobase (the minimal current eliciting at least one action potential) and the first spike latency (depolarizing time before the first spike obtained under rheobase current; Figure 1B). In order to measure the effect of Kv1 channels on the ramp-and-delay phenotype, the depolarizing slope before the first action potential was measured on the 10 ms before the spike threshold [13] (Figure 1B). The spike threshold was measured on phase plots as previously shown [14]. Both slopes and spike thresholds were measured on spikes evoked in the first 200 ms after the current pulse onset to maximize the role of Kv1.1 channels.

Sensitivity of intrinsic excitability to the selective Kv1.1-channel blocker, dendrotoxin-K (DTx-k), was determined by current-clamp recording before and after 5 min of bath application of DTx-K (100 nM). Acquisition was performed at 10 kHz with pClamp10 (Axon Instruments, Molecular Devices, San Jose, CA, USA).

Data were analyzed with ClampFit (Axon Instruments), LabView (National Instruments, Austin, TX, USA) and IgorPro (Wavemetrics, Lake Oswego, OR, USA). Pooled data are presented as mean ± SE and statistical analysis was performed using the Mann–Whitney U test or Wilcoxon rank signed test.

## 3. Results

### 3.1. LRR-mAbs, but Not EPTP-mAbs, Increase Intrinsic Excitability in CA3 Pyramidal Neurons

**Figure 2:**
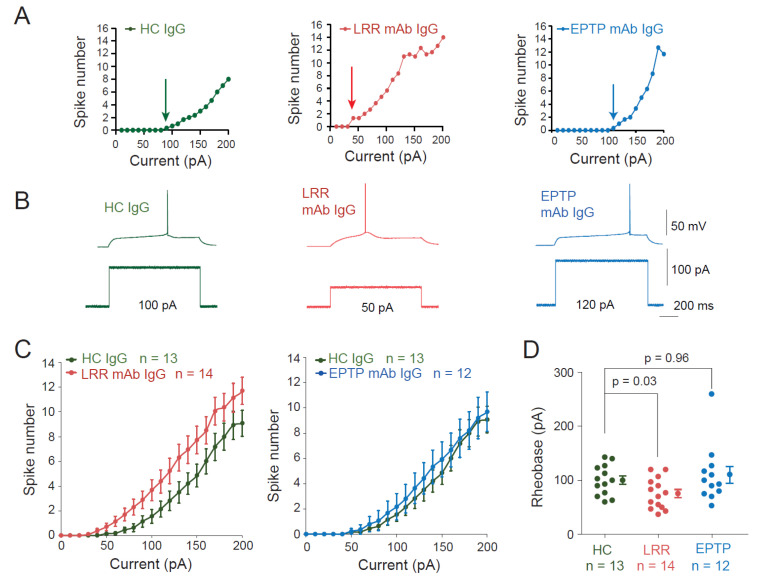
Application of patient-derived LRR-mAb increases intrinsic excitability of CA3 neurons. (**A**) Input–output curves of representative neurons from neurons in rat hippocampal organotypic cultures treated with HC-IgGs (green, left), LRR-mAbs (red, middle), and EPTP-mAbs (blue, right). Arrows indicate the rheobase current. (**B**) Corresponding traces illustrating the rheobase current. (**C**) Averaged input–output curves of LRR- and EPTP-mAbs were compared to control HC-IgGs. Note the leftward shift for LRR- but not for EPTP-mAbs. (**D**) Group data of the rheobase are current for each condition. Error bars represent SEM.

To assess the effect of the two mAbs on neuronal excitability, we performed current-clamp recordings from CA3 pyramidal neurons in organotypic cultures of rat hippocampus treated with the different Abs. Input–output curves were established for each neuron (Figure 2A). A significant decrease in the rheobase was observed after treatment with the LRR-mAb (HC IgGs: 100.1 ± 7.8 pA *n* = 13 vs. LRR-mAb: 75.4 ± 7.5 pA *n* = 14, Mann-Whitney, *p* = 0.035; Figure 2A–D) but not with the EPTP-mAb (HC IgGs: 100.1 ± 7.8 pA *n* = 13 vs. EPTP-mAb: 110.2 ± 15.6 pA *n* = 12, Mann-Whitney, *p* = 0.96; Figure 2A–D). In addition, CA3 neurons treated with the LRR-mAb showed a leftward shift of the input-output curve compared to neurons treated with HC IgGs, whereas input–output curves were not different between neurons treated with the EPTP-mAb and HC IgGs (Figure 2C). Furthermore, no differences in resting membrane potential and passive properties of recorded neurons were observed in neurons treated with LRR-mAbs and EPTP-mAbs compared to those treated with HC-IgGs (resting membrane potential: −58.5 ± 1.2 mV, *n* = 13 in HC-IgG-treated neurons, −58.9 ± 1.8 mV, *n* = 14 in LRR-mAb-treated neurons, and −61.2 ± 1.5 mV, *n* = 12 in EPTP-mAb-treated neurons; Mann–Whitney, *p* = 0.94 and 0.29, respectively; input resistance: 226 ± 7 MΩ, *n* = 13 in HC-IgG-treated neurons vs. 222 ± 13 MΩ, *n* = 14 in LRR-mAb-treated neurons and 222 ± 14 MΩ, *n* = 12 in EPTP-mAb-treated neurons; Mann–Whitney, *p* = 0.45 and 0, 72; capacitance: 338 ± 14 pF *n* = 13 in HC-IgG-treated neurons vs. 330 ± 20 pF *n* = 14 in LRR-mAb-treated cells; Mann–Whitney, *p* = 0.68, and 353 ± 33 pF, *n* = 12 in EPTP-mAb-treated neurons; *p* = 0.89).

**Figure 3:**
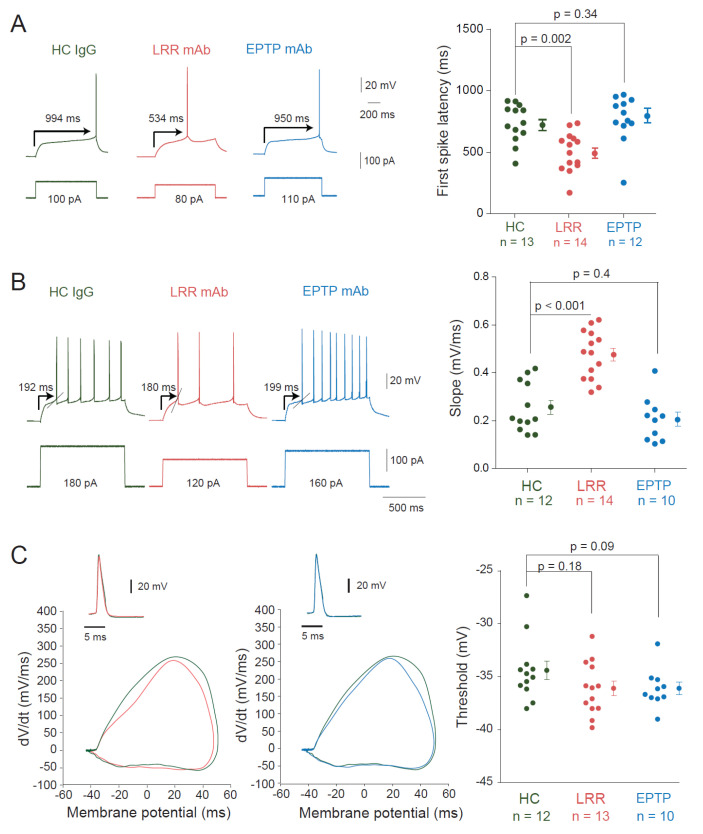
Changes in the spike latency and slope in neurons treated with LRR-mAbs. (**A**) Left, representative traces at the rheobase of neurons treated with HC IgGs (green), LRR-mAbs (red), and EPTP-mAbs (blue). Arrows indicate the latency to the first spike. Right, graph of the latency to the first spike at the rheobase current for each condition. (**B**) Left, the slope of the membrane potential trajectory before the AP is illustrated by the oblique line in the traces (**left**) and the values were reported in the graph (**right**). (**C**) Unchanged spike threshold. Left, comparison of action potential phase plots in LRR- (red) and HC- (green) treated neurons. Middle, comparison of action potential phase plots in EPTP- (blue) and HC- (green) treated neurons. Right, group data. Statistics were performed using Mann–Whitney U-test.

Recordings of HC IgGs-treated neurons showed a characteristic ramp-and-delay profile preceding the evoked spike, strikingly observed at rheobase current (Figure 3A). This feature, also found in untreated control neurons, was characterized as the hallmark of fast-activating slow offset conductance of the D-type current carried by the voltage-gated potassium Kv1 channel [13]. Interestingly, this profile was replaced by a rapid depolarization eliciting the first spike in CA3 neurons treated with the LRR-mAb (Figure 3A). To evaluate this difference, we measured the latency to the first spike. CA3 neurons treated with the LRR-mAb showed a reduced first spike latency compared to HC IgGs treated neurons (494 ± 42 ms *n* = 14 vs. 728 ± 43 ms *n* = 13, Mann–Whitney, *p* = 0.002; Figure 3A). However, no significant difference was observed between neurons treated with HC IgGs or EPTP-mAb (728 ± 43 ms *n* = 13 vs. 762 ± 57 ms *n* = 12, Mann–Whitney, *p* = 0.34; Figure 3A). In addition, the slope before the action potential was found to be faster in neurons treated with LRR-mAb compared to HC IgGs (0.47 ± 0.03 mV/ms, *n* = 14 vs. 0.25 ± 0.03 mV/ms, *n* = 12; MW *p* < 0.001; Figure 3B). However, no difference in the slope was observed between EPTP-mAb and HC IgGs (0.21 ± 0.03 mV/ms, *n* = 10 vs. 0.25 ± 0.03 mV/ms, *n* = 12; MW *p* = 0.4; Figure 3B). Taken together, these results indicate that neurons treated with LRR-mAb display a functional increase in intrinsic excitability, but not those treated with EPTP-mAb. Surprisingly, no significant hyperpolarization of action potential threshold was found in LRR-mAb-treated neurons (−36.1 ± 1.2 mV in LRR, *n* = 13 vs. −34.4 ± 0.8 mV in HC, *n* = 12; MW *p* = 0.18; Figure 3C).

### 3.2. LRR-mAbs Increase Intrinsic Excitability through the Modulation of Kv1 Channels

**Figure 4:**
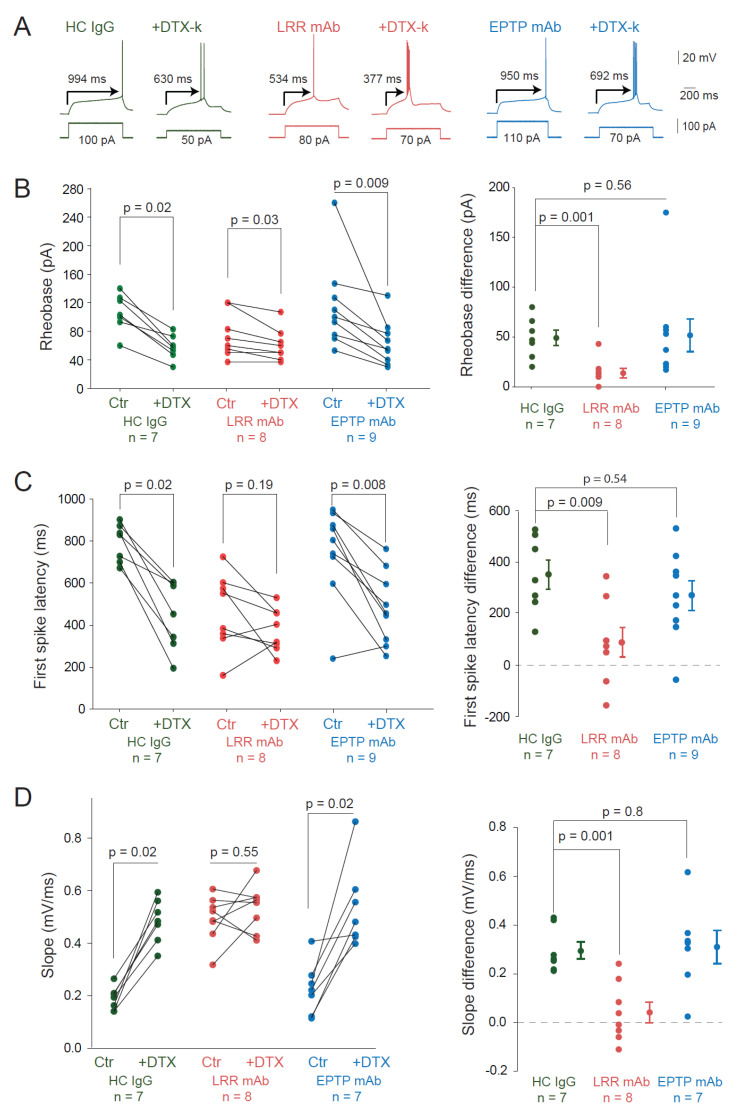
LRR-induced increase in intrinsic excitability is mediated by a decrease of Kv1.1 current. (**A**) The effect of DTx-k on the rheobase of neurons treated with HC IgGs (green), LRR-mAb (red), and EPTP-mAb (blue) was illustrated by the traces at the rheobase current and (**B**, **left**) the graphs of the rheobase current (before and after bath application of DTx-k (100 nM). (**B**, **right**) The effect of DTx-k was highlighted by the subtraction of the rheobase after adding DTx-k to the rheobase before DTx-k, after each antibody treatment. In the same way, the effect of DTx-k on the latency to the first spike at the rheobase current was indicated by (**A**) the arrows and reported in (**C**) the graphs, and the slope of the membrane potential trajectory before the AP was shown in (**D**) the graphs. Error bars represent SEM. Statistical analysis was performed using (**B**–**D left**) the Wilcoxon rank-signed test and the (**B**–**D right**) Mann–Whitney test.

Based on these findings, we asked whether changes in the D-type potassium current could account for the change in intrinsic excitability by measuring the sensitivity to DTx-k after LRR-mAb treatment. DTx-k strongly reduced the rheobase in HC IgG-treated neurons (rheobase = 107 ± 10 pA vs. 58 ± 6 pA *n* = 7, Wilcoxon, *p* = 0.016; Figure 4A,B) and in neurons treated with the EPTP-mAb (rheobase = 115 ± 23 pA vs. 63 ± 12 pA *n* = 9, Wilcoxon, *p* = 0.009; Figure 4A,B), but it had a modest effect in neurons treated with the LRR-mAb (74 ± 11 pA vs. 61 ± 8 pA *n* = 8, Wilcoxon, *p* = 0.035; Figure 4A,B).

To confirm this, we compared the rheobase difference induced by DTx-k in the three cases. Whereas no significant difference was observed between neurons treated with HC IgGs and EPTP-mAb (49 ± 8 pA *n* = 7 vs. 52 ± 16 pA *n* = 9; MW, *p* = 0.56; Figure 4B), we found a fivefold decrease in the rheobase difference in neurons treated with the LRR-mAb compared to HC IgGs (14 ± 5 pA *n* = 8 vs. 49 ± 8 pA *n* = 7, Mann–Whitney, *p* = 0.001; Figure 4B). Moreover, DTx-k induced a loss of the ramp-and-delay phenotype characterized by both the first spike latency and the depolarizing slope before the action potential in neurons treated with HC IgGs or EPTP-mAbs but not in neurons treated with the LRR-mAb (Figure 4C,D). DTx-k significantly reduced the first spike latency in neurons treated with HC IgGs (from 794 ± 34 to 443 ± 62 ms, *n* = 7, Wilcoxon test *p* = 0.016) and EPTP-mAbs (from 754 ± 74 to 485 ± 58 ms, *n* = 9, Wilcoxon test *p* = 0.008) but not in LRR-mAbs (from 462 ± 64 to 376 ± 36 ms, *n* = 8; Wilcoxon test *p* = 0.19; Figure 4C). Similarly, DTx-k significantly enhanced the slope of the depolarizing ramp in neurons treated with HC IgGs (from 0.19 ± 0.02 to 0.48 ± 0.03 mV/ms, *n* = 7; Wilcoxon test *p* = 0.016) and EPTP-mAbs (from 0.23 ± 0.04 to 0.54 ± 0.06 mV/ms, *n* = 7; Wilcoxon test *p* = 0.016) but not in LRR-mAbs (from 0.49 ± 0.03 to 0.54 ± 0.03 mV/ms, *n* = 8; Wilcoxon test *p* = 0.55; Figure 4D). Taken together, these data confirm that a loss of Kv1.1-mediated current induced by the LRR-mAb contributes significantly to the observed increase in intrinsic excitability in CA3 pyramidal neurons.

## 4. Discussion

We show here that two patient-derived mAbs directed against the LRR and EPTP epitopes of LGI1 differentially perturb neuronal excitability in CA3 pyramidal neurons from rat hippocampal organotypic cultures. Compared to IgGs from control patients, LRR-mAbs but not EPTP-mAbs were found to increase neuronal intrinsic excitability. In fact, the input–output curves of LRR-mAb-treated neurons were shifted to the left due to a lower rheobase current. The ramp-and-delay phenotype that is a hallmark of CA3 pyramidal neurons disappeared in LRR-mAb-treated neurons. Finally, treatment with the LRR-mAb, but not the EPTP-mAb, prevented the effect of DTx-k, a blocker of the Kv1.1 voltage-gated potassium channel, indicating that LRR-mAb already reduced functional Kv1.1 channels.

Our results were obtained after 4–5 days of anti-LGI1 application at a concentration of 4 ng/µL. In previous studies, acute application (up to 8 h) [4] or long-lasting application of LGI1 antibodies (14 days) [5] have been shown to produce similar functional effects, suggesting that the application time is not a critical factor.

The increase in excitability observed in LRR-mAb-treated neurons is associated with a lower rheobase, a lower first spike latency, and an elevated depolarizing slope before the action potential. All these changes are mediated by Kv1.1 channels as the application of the selective Kv1.1 channel blocker, DTx-k had little effect on these parameters in LRR-mAb-treated neurons, but not in HC- and EPTP-mAb-treated neurons. The rheobase of LRR-mAb-treated neurons was slightly reduced in DTx-k, indicating that not all Kv1.1 channels are internalized by LRR-mAbs. Surprisingly, no significant change in action potential threshold was observed following treatment with LRR mAbs in our study. As the spike threshold depends on both voltage-gated sodium (Nav) and potassium channels, the lack of spike threshold change may be attributed to the homeostatic reduction of Nav channel density in LRR-mAb-treated neurons, as previously observed in over-excited neurons [15].

Our results are compatible with previous studies showing that LRR-mAbs promote LGI1-ADAM complex internalization [8], which may lead to a reduction in Kv1 channel expression at the cell membrane. This mechanism is further supported by the genetic deletion of LGI1, which decreases both Kv1.1 channel density and D-type currents by more than 50% [16]. As Kv1.1 channels are expressed at both the AIS and presynaptic terminals of CA3 pyramidal neurons [17], the putative consequence of the decrease in Kv1.1-mediated current is an elevated neuronal excitability and an elevated glutamate release, which both concur to promote synchronous epileptiform discharges. Our results were obtained in vitro, as most results describing the effects of LGI1 manipulation on neuronal excitability. It would be nevertheless important to verify that they are confirmed in an in vivo preparation [18].

Antibodies against LGI1 are known to prevent long-term synaptic potentiation (LTP) and memory formation in vivo [5,8]. Interestingly, while both LRR-mAbs and EPTP-mAbs equally prevent LTP, only LRR-mAbs impair object recognition [8], suggesting that object recognition may involve mechanisms different from LTP. However, the lack of LTP in animals treated with LGI1-Abs is difficult to interpret as epileptiform activity itself triggers synaptic potentiation that prevents further LTP induction [19]. As epileptiform activity is observed in animals treated with LGI1-Abs, there are also good reasons to believe that the lack of LTP observed in these animals is in fact due to the epilepsy-induced occlusion of LTP [20].

The molecular mechanism by which EPTP-mAbs mediate their effect is likely to occur through the prevention of LGI1 binding to its native receptors. As suggested in a recent study [21], EPTP-mAb (mAb6212) could bind LGI1 in non-neuronal complexes such as glial cells (astrocytes and oligodendrocytes) that may thus explain the lack of excitability regulation induced by this particular EPTP-mAb. Further experiments would be needed to elucidate these questions.

## Data Availability

All data reported in this study are included in the manuscript.

## References

[1] Irani S.R., Michell A.W., Lang B., Pettingill P., Waters P., Johnson M.R., Schott J.M., Armstrong R.J.E., Zagami A.S., Bleasel A. (2011). Faciobrachial Dystonic Seizures Precede Lgi1 Antibody Limbic Encephalitis. Ann Neurol..

[2] Aurangzeb S., Symmonds M., Knight R., Kennett R., Wehner T., Irani S. (2017). LGI1-Antibody Encephalitis Is Characterised by Frequent, Multifocal Clinical and Subclinical Seizures. Seizure.

[3] Thompson J., Bi M., Murchison A.G., Makuch M., Bien C.G., Chu K., Farooque P., Gelfand J.M., Geschwind M.D., Hirsch L.J. (2018). The Importance of Early Immunotherapy in Patients with Faciobrachial Dystonic Seizures. Brain.

[4] Lalic T., Pettingill P., Vincent A., Capogna M. (2011). Human Limbic Encephalitis Serum Enhances Hippocampal Mossy Fiber-CA3 Pyramidal Cell Synaptic Transmission: Limbic Encephalitis in the Hippocampus. Epilepsia.

[5] Petit-Pedrol M., Sell J., Planagumà J., Mannara F., Radosevic M., Haselmann H., Ceanga M., Sabater L., Spatola M., Soto D. (2018). LGI1 Antibodies Alter Kv1.1 and AMPA Receptors Changing Synaptic Excitability, Plasticity and Memory. Brain.

[6] Yamagata A., Fukai S. (2020). Insights into the Mechanisms of Epilepsy from Structural Biology of LGI1–ADAM22. Cell. Mol. Life Sci..

[7] Kornau H.-C., Kreye J., Stumpf A., Fukata Y., Parthier D., Sammons R.P., Imbrosci B., Kurpjuweit S., Kowski A.B., Fukata M. (2020). Human Cerebrospinal Fluid Monoclonal LGI1 Autoantibodies Increase Neuronal Excitability. Ann. Neurol..

[8] Ramberger M., Berretta A., Tan J.M.M., Sun B., Michael S., Yeo T., Theorell J., Bashford-Rogers R., Paneva S., O’Dowd V. (2020). Distinctive Binding Properties of Human Monoclonal LGI1 Autoantibodies Determine Pathogenic Mechanisms. Brain.

[9] Ohkawa T., Fukata Y., Yamasaki M., Miyazaki T., Yokoi N., Takashima H., Watanabe M., Watanabe O., Fukata M. (2013). Autoantibodies to Epilepsy-Related LGI1 in Limbic Encephalitis Neutralize LGI1-ADAM22 Interaction and Reduce Synaptic AMPA Receptors. J. Neurosci..

[10] Schulte U., Thumfart J.-O., Klöcker N., Sailer C.A., Bildl W., Biniossek M., Dehn D., Deller T., Eble S., Abbass K. (2006). The Epilepsy-Linked Lgi1 Protein Assembles into Presynaptic Kv1 Channels and Inhibits Inactivation by Kvβ1. Neuron.

[11] Rama S., Zbili M., Fékété A., Tapia M., Benitez M.J., Boumedine N., Garrido J.J., Debanne D. (2017). The Role of Axonal Kv1 Channels in CA3 Pyramidal Cell Excitability. Sci. Rep..

[12] Debanne D., Boudkkazi S., Campanac E., Cudmore R.H., Giraud P., Fronzaroli-Molinieres L., Carlier E., Caillard O. (2008). Paired-Recordings from Synaptically Coupled Cortical and Hippocampal Neurons in Acute and Cultured Brain Slices. Nat. Protoc..

[13] Cudmore R.H., Fronzaroli-Molinieres L., Giraud P., Debanne D. (2010). Spike-Time Precision and Network Synchrony Are Controlled by the Homeostatic Regulation of the D-Type Potassium Current. J. Neurosci..

[14] Fékété A., Ankri N., Brette R., Debanne D. (2021). Neural Excitability Increases with Axonal Resistance between Soma and Axon Initial Segment. Proc. Natl. Acad. Sci. USA.

[15] Grubb M.S., Burrone J. (2010). Activity-Dependent Relocation of the Axon Initial Segment Fine-Tunes Neuronal Excitability. Nature.

[16] Seagar M., Russier M., Caillard O., Maulet Y., Fronzaroli-Molinieres L., De San Feliciano M., Boumedine-Guignon N., Rodriguez L., Zbili M., Usseglio F. (2017). LGI1 Tunes Intrinsic Excitability by Regulating the Density of Axonal Kv1 Channels. Proc. Natl. Acad. Sci. USA.

[17] Zbili M., Rama S., Benitez M.-J., Fronzaroli-Molinieres L., Bialowas A., Boumedine-Guignon N., Garrido J.J., Debanne D. (2021). Homeostatic Regulation of Axonal Kv1.1 Channels Accounts for Both Synaptic and Intrinsic Modifications in the Hippocampal CA3 Circuit. Proc. Natl. Acad. Sci. USA.

[18] Baudin P., Whitmarsh S., Cousyn L., Roussel D., Lecas S., Lehongre K., Charpier S., Mahon S., Navarro V. (2022). Kv1.1 Channels Inhibition in the Rat Motor Cortex Recapitulates Seizures Associated with Anti-LGI1 Encephalitis. Prog. Neurobiol..

[19] Debanne D., Thompson S.M., Gähwiler B.H. (2006). A Brief Period of Epileptiform Activity Strengthens Excitatory Synapses in the Rat Hippocampus in Vitro. Epilepsia.

[20] Debanne D., El Far O. (2018). Pre- and Postsynaptic Effects of LGI1 Autoantibodies in a Murine Model of Limbic Encephalitis. Brain.

[21] Ramirez-Franco J., Debreux K., Extremet J., Maulet Y., Belghazi M., Villard C., Sangiardi M., Youssouf F., El Far L., Lévêque C. (2022). Patient-Derived Antibodies Reveal the Subcellular Distribution and Heterogeneous Interactome of LGI1. Brain.

